# Refinements of LC-MS/MS Spectral Counting Statistics Improve Quantification of Low Abundance Proteins

**DOI:** 10.1038/s41598-019-49665-1

**Published:** 2019-09-20

**Authors:** Ha Yun Lee, Eunhee G. Kim, Hye Ryeon Jung, Jin Woo Jung, Han Byeol Kim, Jin Won Cho, Kristine M. Kim, Eugene C. Yi

**Affiliations:** 1 Department of Molecular Medicine and Biopharmaceutical Sciences, Graduate School of Convergence Science and Technology and College of Medicine or College of Pharmacy, Seoul National University, Seoul, 03080 South Korea; 2 Department of Systems Immunology, Division of Biomedical Convergence, College of Biomedical Science, Kangwon National University, Gangwon, 24341 South Korea; 3 Department of Integrated OMICS for Biomedical Science, Graduate School, Yonsei University, Seoul, 03722 South Korea

**Keywords:** Breast cancer, Proteome informatics

## Abstract

Mass spectrometry-based spectral count has been a common choice of label-free proteome quantification due to the simplicity for the sample preparation and data generation. The discriminatory nature of spectral count in the MS data-dependent acquisition, however, inherently introduces the spectral count variation for low-abundance proteins in multiplicative LC-MS/MS analysis, which hampers sensitive proteome quantification. As many low-abundance proteins play important roles in cellular processes, deducing low-abundance proteins in a quantitatively reliable manner greatly expands the depth of biological insights. Here, we implemented the Moment Adjusted Imputation error model in the spectral count refinement as a post PLGEM-STN for improving sensitivity for quantitation of low-abundance proteins by reducing spectral count variability. The statistical framework, automated spectral count refinement by integrating the two statistical tools, was tested with LC-MS/MS datasets of MDA-MB468 breast cancer cells grown under normal and glucose deprivation conditions. We identified about 30% more quantifiable proteins that were found to be low-abundance proteins, which were initially filtered out by the PLGEM-STN analysis. This newly developed statistical framework provides a reliable abundance measurement of low-abundance proteins in the spectral count-based label-free proteome quantification and enabled us to detect low-abundance proteins that could be functionally important in cellular processes.

## Introduction

Quantitative proteome analysis between two or more systems is an indispensable part of functional proteomics as relative abundance of proteins reflects a functional dynamic in biological system^1,2^. Mass spectrometry (MS) has become an important tool in quantitative proteomics method including a stable isotope labeling method *in-vitro* or *in-vivo*^3–7^ and MS spectral counting (MS-SC) method^8^. The MS-SC has been widely used for the label-free quantitative proteomics as it affords simpler and faster sample preparation and data analysis^9^. In the label-free MS method, complex protein mixtures in biological matrices such as plasma, serum, or tissue protein extracts are enzymatically digested to peptides, which results in more complex peptide analytes in several orders of magnitude. The peptide mixtures are then analyzed by liquid chromatography tandem mass spectrometry (LC-MS/MS) in a data-dependent acquisition (DDA) mode. In the DDA mode, peptides are selected and prioritized for MS/MS fragmentation based on their precursor ion signal intensity^10^. Peptide MS/MS spectra are being searched against the relevant protein database and identified. The number of identified redundant peptides are then statistically analyzed for the quantitative proteome changes in the given biological sample matrices using a variety of statistical methods.

Several statistical methods for the peptide SC-based quantitative proteomics were proposed and implemented. There are empirical tests which were developed specifically for the SC quantification^11^, such as the spectral index (SpI)^12^ and QSpec^13^ methods. The statistical tools designed for gene expression microarray have also been used for the analysis of label-free MS proteomics^14^, the significance analysis of microarrays (SAM)^15^ and the normalized spectral abundance factor coupled with Power Law Global Error Model-Signal To Noise (PLGEM-STN) statistics^16^ are two examples. Coupling the normalization method with standard statistical t-test is another way to quantify differentially expressed proteins (DEPs); the SC normalization methods include weighted scoring from peptide match score^17^, normalization by the number of potential peptide matches^18^, peptide sequence length^19^, peptide proteotypicity^20^, and fusion of the probability of identification into counting^21^.

One of the shortcomings of the SC-based label-free proteomics is the inherent bias against low-abundance proteins during the MS/MS data acquisition, which may result in in-sensitive quantification. Due to the discriminatory MS/MS data acquisition, the measured SC of low-abundance proteins (SC mean <5) yield larger SC variation^22–25^ and such SC variation leads to quantitative underestimation on true differences in their expression levels^26^. In this study, we developed the low-abundance protein-centric refinement to quantify them for better sensitivity by implementing the Moment Adjusted Imputation (MAI) error model. The MAI model adjusts the mis-measured data that result from device-related error or biological fluctuations, reflecting the latent variable distribution, which in turn improves statistical parameter estimation^27,28^. We applied the model in normalizing SC, and the refined SC was then applied to PLGEM-STN statistical analysis^14^. The MAI model in conjunction with PLGEM-STN tool reduces the variation of SC between replicate analyses, thus enhancing the validity of p-values for the low-abundance proteins. This combined statistical approach was validated by MDA-MB468 breast cancer (BC) cells grown under high glucose (HG) and glucose deprivation (GD) conditions. We obtained about 30% more quantifiable proteins with confident cut off p-value (<0.03). The majority of proteins were found to be endogenously low expressed and involved in important biological roles in the given cellular conditions. The SC refinement via MAI method results in additional identification of DEPs with better sensitivity and is generally applicable for the in-depth proteome analysis.

## Results

### PLGEM-STN analysis

**Figure 1:**
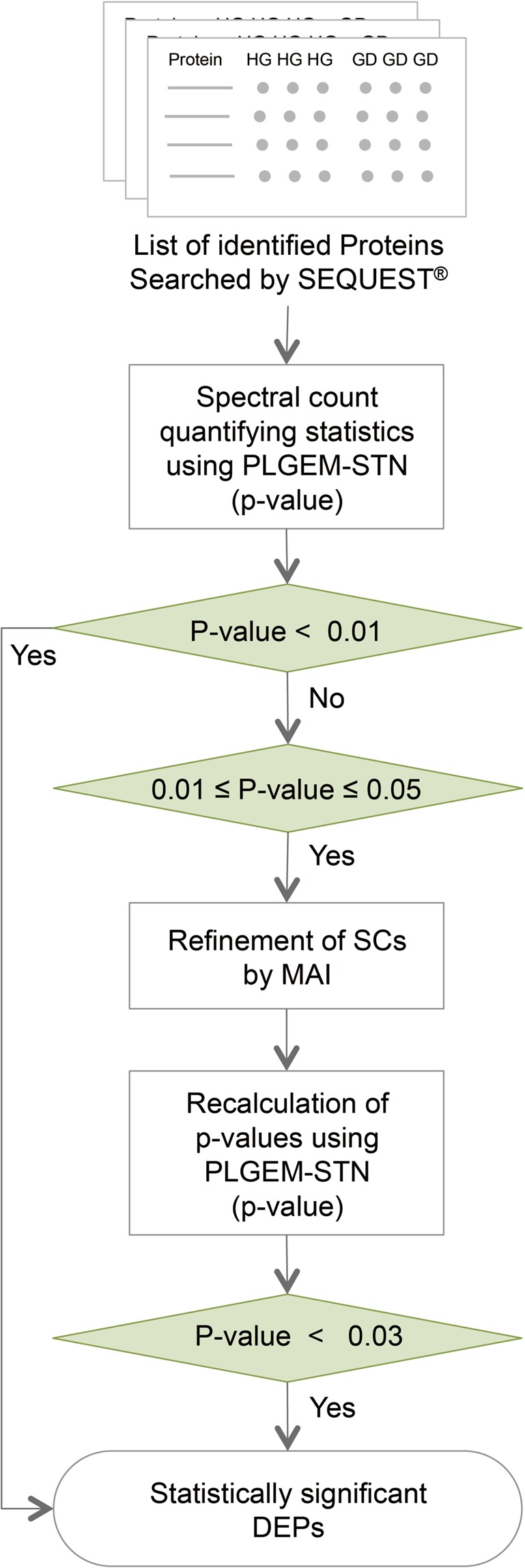
The overall scheme of the SC refinement. The triplicate datasets of SC were analyzed by PLGEM-STN and confident DEPs were selected with p-value threshold less than 0.01. The further quantification refinement was performed for the proteins within 0.01 ≤ p-value ≤ 0.05 using MAI estimators. The proteins with recalculated p-value < 0.03 were considered statistically significant and were combined with DEPs of p-value < 0.01.

We used the test sample matrix of nuclear and cytoplasm proteins of MDA-MB468 BC cells grown under HG and GD conditions. SDS-PAGE was used to fractionate the nuclear and cytoplasm proteins prior to LC-MS/MS analysis to identify proteins over a wider dynamic range thereby increasing the detection of low-abundance proteins. After in-gel digestion of proteins, LC-MS/MS analysis of extracted peptides followed by protein sequence database searching, we identified a total of 2,525 proteins (at least two unique peptides with false discovery rate (FDR)≤0.1%) (Supplementary Table S1). We performed the PLGEM-STN analysis on 2,525 identified proteins, and quantified 681 DEPs (Supplementary Table S2) with p-value threshold less than 0.01, which is a typical p-value threshold for statistical significance. While the PLGEM-STN analysis provides statistical confidence signal-to-noise ratio (p-value < 0.01) for high-abundance proteins in the label-free quantitation, the majority of low-abundance proteins (SC mean <5) suffer from the statistical confidence levels due to their SC variations. To improve quantitation sensitivity of low-abundance proteins, we statistically refined SC of proteins within PLGEM-STN p-value ≥ 0.01 and ≤0.05 to identify proteins that were quantitatively underestimated the true differences in their expression levels (Fig. 1).

### Spectral count variation and PLGEM-STN p-values

**Figure 2:**
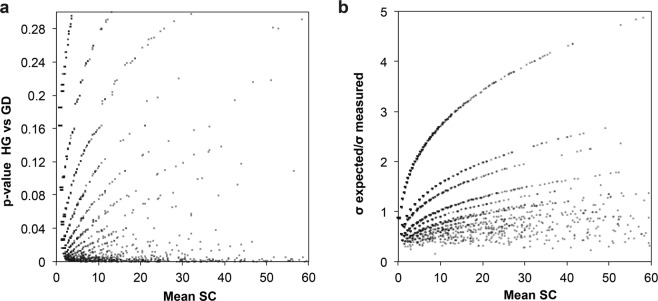
Relationship between the number of SC and PLGEM-STN statistical factors. (**a**) A plot of PLGEM-STN p-values and the mean values of triplicate SC of MDA-MB468 cells grown under HG and GD conditions. The plot demonstrates that proteins with low SC (SC mean < 5) have higher p-values (average 0.2293) and proteins with high SC (SC mean > 100) have lower p-values (average 0.0983). (**b**) A plot of measured standard deviation over expected standard deviation (σ_expected/σ_measured) and the mean of SC. The plot demonstrates that proteins with low SC tend to have more differences between expected standard deviation and measured standard deviation.

To observe the distribution of PLGEM-STN p-values over SC numbers of low-abundance proteins, we plotted the PLGEM-STN p-values of identified proteins over the mean values of SCs and observed that the mean p-values of proteins identified with lower SC (SC mean <5) was about 0.2293, whereas proteins identified with higher SC (SC mean ≥5) showed the mean p-values around 0.0983 (Fig. 2a). Furthermore, we plotted the ratio of expected and measured standard deviations (σ-expected/σ-measured) over the mean of repeated SC detection showing that low-abundance proteins have poor reproducibility on SC during the triplicate analysis compared with high-abundance proteins (Fig. 2b). We assumed the σ-expected as standard deviation calculated from the PLGEM linear regression model, which explicitly assume a constant coefficient of variation (CV) and deriving standard deviation varying proportionally with the mean. About 44% (636 out of 1,445) proteins with low SC (SC mean <5) had σ-measured greater than σ-expected, σ-expected/σ-measured <1, whereas 37% (391 out of 1,065) proteins with mean of SC ≥5 had σ-expected/σ-measured <1. This observation indicated that LC-MS/MS data acquisition of those low-abundance proteins have poor reproducibility in spectral counting.

### MAI statistical analysis of low-spectral count proteins

Proteins, scoring PLGEM-STN p-values between 0.01 and 0.05, were statistically refined to improve their quantitative confidence p-values using MAI estimator^27^. We implemented the MAI to the triplicate breast cancer SC datasets to identify proteins that were initially filtered out by the PLGEM-STN statistics. The mis-measured observation is *W*_*i*_ and true values is *X*_*i*_ for latent variables for *i* = 1,…,*n*. The objective of the MAI is to construct adjusted value of the *W*_*i*_ using recreated true value $\widehat{{X}_{i}}$ where $\widehat{{X}_{i}}$ are unbiased sample moment estimates of the corresponding moment of *X*_*i*_, a point where $E({n}^{-1}{\sum }_{i=1}^{n}\widehat{{X}_{i}^{r}})=E({X}^{r})$, *r* = 1, …, *M*. The adjusted $\widehat{\,{X}_{i}}$ that we would like to estimate are obtained by minimizing ${\sum }_{i=1}^{n}{({W}_{i}-{X}_{i})}^{2}$ subject to constraints on the moments and cross-products. This allows the MAI estimator to be defined in the following function

1 $$\widehat{{X}_{i}}={W}_{i}\hat{a}+\bar{W}(1-\hat{a})$$

where $\bar{W}={n}^{-1}{{\sum }_{i=1}^{n}W}_{i}$, $\hat{a}={({\hat{\sigma }}_{x}^{2}/{\hat{\sigma }}_{w}^{2})}^{1/2}$, ${\hat{\sigma }}_{w}^{2}={n}^{-1}{\sum }_{i=1}^{n}{({W}_{i}-\bar{W})}^{2}$ and ${\hat{\sigma }}_{x}^{2}\,$ = est of ${\sigma }_{x}^{2}$ by PLGEM from the linear regression model; $\mathrm{ln}(s)=k\,\mathrm{ln}(\bar{x})+c+\varepsilon$ (*s* and $\bar{x}$ as standard deviation and mean of repeated measures, $k$ as slope, *c* as intercept and *ε* as error term). We have considered the $\widehat{{X}_{i}}$ as the refined SCs, *W*_*i*_ as measured SCs, *i* as repeated number of measurements in LC-MS/MS, and $\hat{a}$ as relation between measured variable *σ*_*w*_ and potentially error-free covariate *σ*_*x*_ $({({\widehat{{\sigma }_{x}}}^{2}/{\widehat{{\sigma }_{w}}}^{2})}^{1/2})$.

This adjustment of SC using the MAI was made when the plot of triplicated SC data exhibit skewness. The triplicated SC with skewness greater than 0 was regarded to be overestimated, skewness less than 0 to be underestimated and skewness equal to 0 to be truly estimated. We assume that *W*_1_ ≤ *W*_2_≤…≤*W*_*n*_ and ${X}_{1}\le {X}_{2}\le \ldots \le {X}_{n}$. For two different conditions, the objective function is

$$\begin{array}{c}{\rm{skewness}}\,{\rm{of}}\,W > 0,\,\,\widehat{{X}_{n}}={W}_{n}\hat{a}+\bar{W}(1-\hat{a})\,{\rm{and}}\,\,({X}_{1},\ldots ,{X}_{n-1})=({W}_{1},\ldots ,{W}_{n-1})\\ \,{\rm{skewness}}\,{\rm{of}}\,W < 0,\,\widehat{{X}_{1}}={W}_{1}\hat{a}+\bar{W}(1-\hat{a})\,{\rm{and}}\,\,({X}_{2},\ldots ,{X}_{n-1})=({W}_{2},\ldots ,{W}_{n-1})\end{array}$$

Using the MAI estimator values, DEPs were identified through PLGEM-STN once again and considered proteins with p-value < 0.03 as statistically significant DEPs.

**Figure 3:**
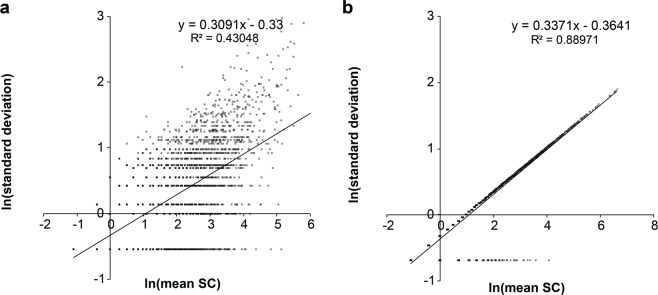
Relationship between the mean of SC and standard deviation after the MAI refinement. (**a**) A plot of standard deviation and average of triplicate SC in log scale showing a regression line y = 0.3091x-0.33, R^2^ = 0.4305. (**b**) A plot of standard deviation and average in log scale after the MAI refinement showing a regression line y = 0.3371x-0.36, R^2^ = 0.8897.

**Figure 4:**
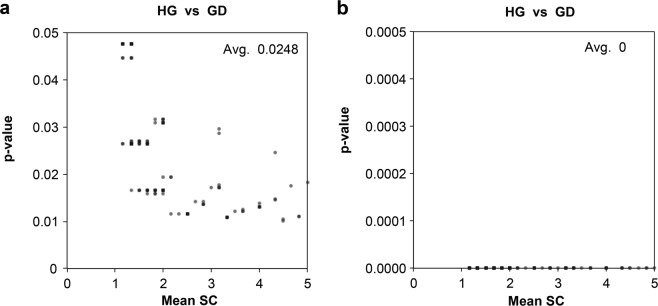
Relationship between the mean of SC and p-values after the MAI refinement. (**a**) A plot of p-values over mean SC of low-abundance before the refinement (**b**) after the refinement.

After normalizing the SC numbers using the MAI, we identified additional 279 DEPs within the range of confident cut-off values (p-value < 0.03) (Supplementary Table S2). We plotted the log scale of standard deviation over the mean of triplicate SC using 960 DEPs (681 DEPs with p-value < 0.01 and 279 MAI refined DEPs) and showed that the MAI normalized SC according to the regression (Fig. 3a,b), the proteins were aligned along the linear line (from R^2^ value 0.43048 to 0.88971). The observation showed that via the normalization of SC, variations were decreased to the standard deviation computed by PLGEM-STN. To validate the decrease in p-values, we plotted p-values over the mean of SC of low-abundance proteins after the MAI refinement. The average of p-values decreased to 0 (Fig. 4b) from the previous average p-values, 0.0248 (Fig. 4a). These results demonstrated that the MAI improved reproducibility and led to a decrease in p-values. The R script for the automated calculation of SC refinement using the MAI is included in the Supplementary Information S1.

### Protein-protein interaction network analysis of DEPs

**Figure 5:**
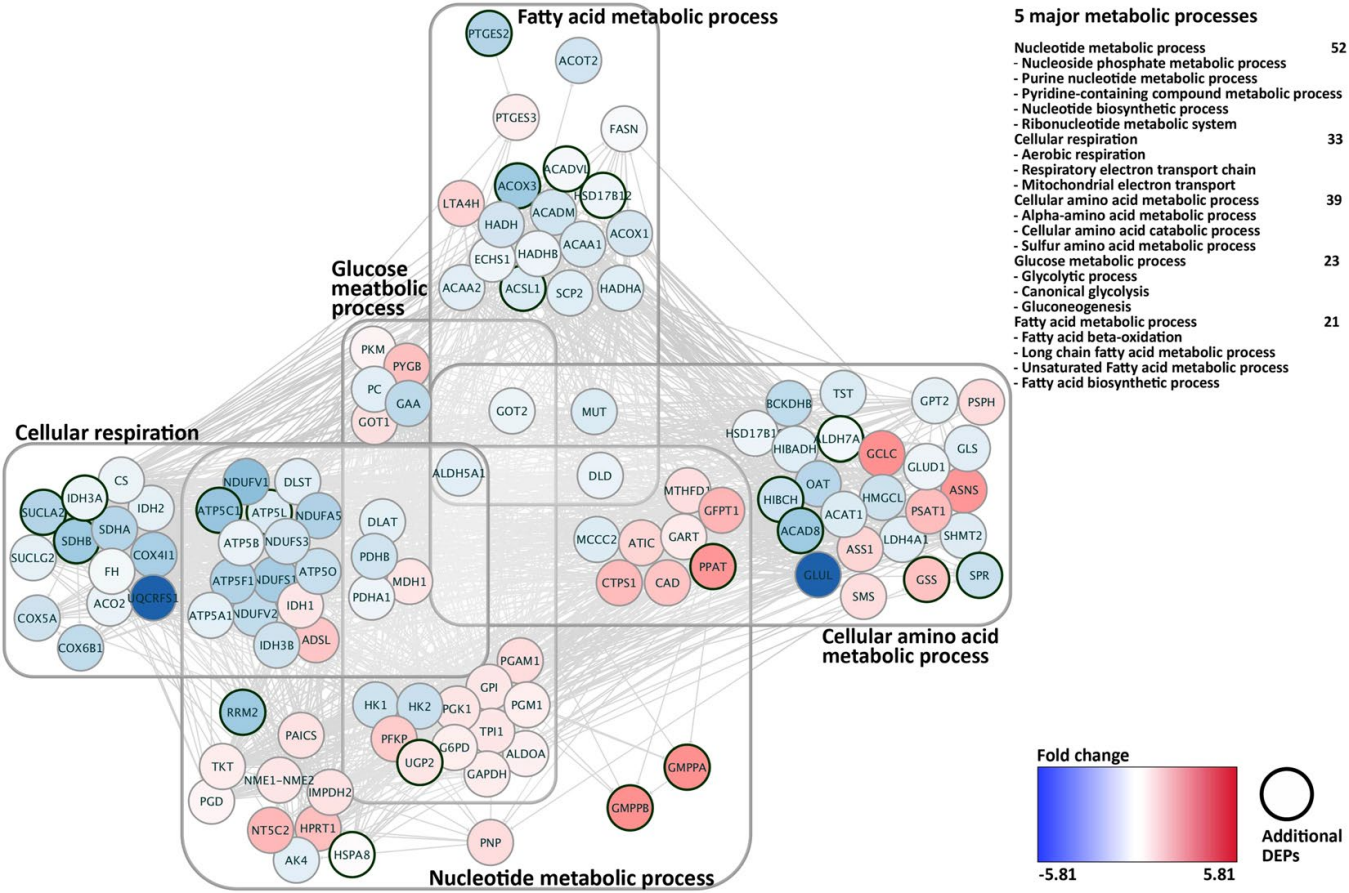
PPI network of DEPs between HG vs GD conditions in breast cancer. Constructed PPI network consists of 5 metabolic processes (nucleotide metabolic process, cellular respiration, cellular amino acid metabolic process, glucose metabolic process and fatty acid metabolic process) with 93 DEPs.

To assess the involvement of the MAI-refined DEPs in the GD and HG breast cancer functional profile, we combined all DEPs identified from both PLGEM-STN and MAI analyses and constructed the Protein-Protein Interaction (PPI) using STRING^29^ and Cytoscape^30^. Since the metabolic shift with a concomitant dysfunction of mitochondria respiration is a hallmark in tumor cell^31,32^, we focused on the metabolic pathway from the PPI map. As embedded newly identified DEPs (bold circles) to the PPI network of 681 DEPs (PLGEM p-value < 0.01) (Fig. 5), the fatty acid metabolic process was enriched by down-regulated MAI-refined DEPs (ACOX3, PTGES2, HSD17B12, ACADVL, and ACSL1) and the cellular respiration was enriched by down-regulated SUCLA2, SDHB, IDH3A, ATP5C1, and ATP5L. The added regulatory MAI-refined DEPs implicated that the metabolic shift with a concomitant dysfunction of mitochondria respiration with the down-regulation on fatty acid metabolism, which is the hallmark in the BC subtype^32^. Five MAI-refined DEPs (ALDH7A, HIBCH, ACAD8, SPR, GSS and PPAT) also enriched the cellular amino acid metabolic process. Glycosylation catalytic enzymes such as GMPPA and GMPPB including UGP2, RRM2 and HSPA8 intensify the nucleotide metabolic process, which is supported by the fact that the cellular system degrades amino acids for energy formation^33^ and deter proliferation activity for energy conservation by down regulating transcription activities under the GD condition^34^.

### Quantitative validation of MAI-refined DEPs

**Figure 6:**
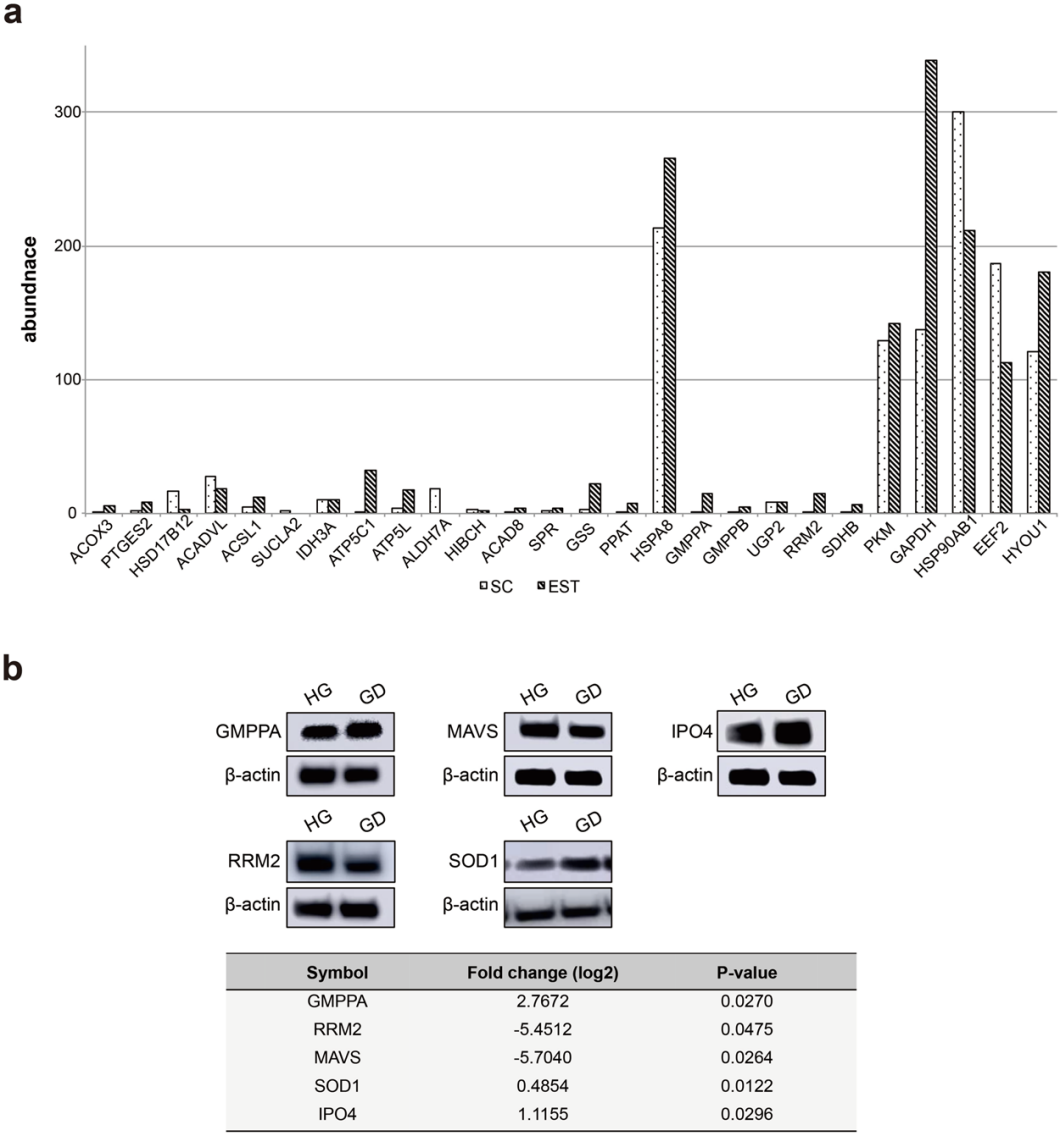
Analysis of expression levels of the MAI-refined DEPs. (**a**) A plot of EST abundances of 21 MAI-refined DEPs (ACOX3, PTGES2, HSD17B12, ACADVL, ACSL1, SUCLA2, IDH3A, ATP5C1, ATP5L, ALDH7A, HIBCH, ACAD8, SPR, GSS, PPAT, HSPA8, GMPPA, GMPPB, UGP2, RRM2 and SDHB) with selected high-abundance DEPs (PKM, GAPDH, HSP90AB1, EEF2 and HYOU1) as a reference group (SC > 100 and PLGEM-STN p-value < 0.01). (**b**) Western blot analysis of GMPPA, RRM2, MAVS, SOD1 and IPO4 expression levels in MDA-MB468 cells grown under the HG and GD conditions and measured relative abundance of GMPPA, RRM2, MAVS, SOD1 and IPO4 calculated from SC. Full-length blots are presented in Supplementary Information S2.

For the cross-validation on the MAI/PLGEM-STN quantitative measurements, we analyzed 279 MAI refined DEPs with the MS1-based quantification using Scaffold Q+. We observed that the correlativity of two different quantitative measurements is about 88% (Supplementary Table S3 and Supplementary Information S3), suggesting a quantitative reliability of the MAI refined DEPs. To further verify whether the MAI-refined DEPs have low predicted expression gene levels, we accessed their gene Expressed Sequence Tag (EST) abundance^35,36^ (Supplementary Information S4). The majority of the MAI-refined DEPs, including ACOX3, PTGES2, HSD17B12, ACADVL, ACSL1, SUCLA2, IDH3A, ATP5C1, ATP5L, ALDH7A, HIBCH, ACAD8, SPR, GSS, PPAT, HSPA8, GMPPA, GMPPB, UGP2, RRM2, and SDHB involved in the metabolic processes as depicted in the PPI network (Fig. 5), are with EST abundance below 35, while proteins measured high SC (SC >100) are with above 100 EST abundance values, demonstrating a positive correlation between SC and EST abundance (Fig. 6a). To verify the results from the MAI-refinement, we performed Western blot analysis to measure the relative expression levels of the MAI-refined proteins involved in the metabolic processes (GMPPA, RRM2 and MAVS), a protein involved in antioxidant activity (SOD1) and a protein transporter (IPO4). We chose these proteins for validation since they are involved in essential functional pathways of cancer cells: GMPPA is glycosylation catalytic enzymes^37^, RRM2 catalyzes the biosynthesis of deoxyribonucleotide^38^, MAVS acts in innate immune defense^39^, and SOD1 regulates the reactive oxygen stress by destroying superoxide radicals^40^. GMPPA, SOD1 and IPO4 showed an elevated expression levels in the GD condition as compared with the HG condition; RRM2 and MAVS were down regulated in the GD condition as comparedwith the HG condition, which showed positive correlation with the SC quantitative readouts (Fig. 6b).

## Discussion

In the SC-based label-free quantitative proteomics, the MS data acquisition via DDA mode is biased towards proteins of high abundance. The discriminatory nature of the DDA mode in the MS data acquisition introduces the inherent variation of SC for endogenously low expressed proteins in the replicate LC-MS/MS analysis. The SC variation hampers the sensitive quantitative measurements for low-abundance proteins, hence, frequently leading to underestimation of their true abundance. As many low-abundance proteins play important roles in many essential cellular processes, deducing low-abundant proteins in a quantitatively reliable manner greatly expands the depth of biological insights.

In this study, we implemented the MAI error model as a post PLGEM-STN analysis to extend the quantification sensitivity and accuracy of the proteins that were identified with PLGEM-STN p-values between 0.01 and 0.05. The MAI is an error model to offset errors associated with device-related error or biological fluctuations. It replaces the mis-measured data with estimators that have asymptotically same distribution as a latent variable of interest up to finite number of moment. Thomas *et al*.^27^ investigated the performance of MAI in logistic regression and demonstrated superior results to the commonly used moment error models such as moment reconstruction (MR)^41^ and regression calibration (RC)^42,43^. The RC, a most commonly applied measurement error model, works most effectively in correction for linear model covariates with minor measurement error, while the MR, a model explored from a Bayesian perspective, is known to work best at normally distributed re-constructed true values. The MAI not only retains the convenience of other imputation methods, but also enables incorporation of a variety of distribution^27^. Therefore, we implemented the MAI model in SC refinement to generate reduced SC variability of low-abundance proteins and to improve sensitivity in quantification. To ensure a consistent evaluation of workflow that can also be used by others, we developed an R script that includes all automated calculation of refined SCs using the MAI error model (Supplementary Information S1).

We demonstrated the MAI error model with a subset of identified protein groups (PLGEM-STN p-value ≤ 0.05) obtained from the label-free semi-quantitative proteomics study of MDA-MB468 BC cells grown under HG and GD conditions. We quantitatively analyzed the expression of 2,525 proteins between the two conditions and identified 681 DEPs with PLGEM-STN p-value less than 0.01. Proteins within p-value ≥ 0.01 and ≤0.05 were refined in their SC by the MAI error model to improve the performance of SC-based label-free experiment in quantifying low-abundance proteins. After the MAI statistics, the p-values were recomputed and additional 279 proteins were quantified with confident cut off p-value less than 0.03, which were further confirmed of their statistical validity by the MS1-based quantification. Some of these quantitatively refined proteins (ACOX3, PTGES2, HSD17B12, ACADVL, ACSL1, SUCLA2, IDH3A, ATP5C1, ATP5L, ALDH7A, HIBCH, ACAD8, SPR, GSS, PPAT, HSPA8, GMPPA, GMPPB, UGP2, RRM2 and SDHB) enriched five major PPI networks including nucleotide metabolic process, cellular respiration, cellular amino acid metabolic process, glucose metabolic process, and fatty acid metabolic process. To validate whether the quantitatively rescued proteins are intrinsically low at the genomic levels, we further compared their relative expressions (based on SC values) with the number of EST DNA sequence reads. Notably, the expression levels of proteins positively correlated with EST DNA sequence reads in BC patients, and most of these proteins showed low EST levels (<35) implicating that the MAI-refined DEPs were statistically valid. Furthermore, we validated the changes in expression levels by Western blotting. Collectively, the results were supported by the fact that the cellular system degrades amino acids for energy formation^33^ and deter proliferation activity for energy conservation by down regulating transcription activities under the glucose deprivation condition^34^.

SC is still the most widely used label-free MS-based semi-quantitative approach. However, inherent variation in SC for low-abundance proteins holds a limitation in accurate and sensitive proteome quantification, which may hamper the detection of biologically important proteins. More importantly, as proteomics study complements functional genomics study, advancement in quantitative proteomics by enabling more quantitatively accurate and sensitive proteome features as to the dynamic-range of genomics data is essential. We believe the MAI error model as a post PLGEM-STN to the global label-free dataset benefits to this end. We demonstrated that the MAI refinement improved quantification sensitivity and accuracy of proteins in low-abundance as evidenced by additionally quantified DEPs, which were enriched in the major metabolic functional pathways. The ease of use of the MAI error model as a part of the PLGEM-STN analysis would thus enable to quantify low-abundance proteins that could be functionally important in cellular processes.

## Methods

### Cell lysis and in-solution digestion

MDA-MB468 cells were grown at 37 °C in an atmosphere of 5% CO_2_ in DMEM containing 10% FBS (HyClone, Logan, UT, USA) under high glucose (25 mM glucose incubation) or glucose derivation (0 mM glucose incubation) condition for 48 hours. Cells (1 × 10^7^) were washed three times with cold PBS and harvested by centrifugation (500 × *g*, 5 min, 4 °C) with a buffer containing 0.1 mM oxidized GSH (Sigma-Aldrich, St. Louis, MO, USA) in PBS. The cells were lysed with M-per lysis buffer (Thermo Scientific, San Jose, CA, USA) with protease inhibitor (cOmplete; Roche Diagnostics, Mannheim, Germany) and phosphatase inhibitor (Roche Diagnostics, Mannheim, Germany) cocktail, followed by a brief sonication on ice. The cell lysates were centrifuged at 14,000 × *g* for 10 min and collected the supernatant containing nucleus and cytosolic proteins. Concentration of protein was determined using a BCA Protein Assay Kit (Thermo Scientific). Proteins were reduced with 10 mM DTT in 6 M urea and alkylated with 30 mM iodoacetamide. The protein samples were then diluted to 1 M urea with 50 mM ammonium bicarbonate, and trypsin (Promega, Madision, WI, USA) was added at a ratio of 1:50 (trypsin:protein), followed by overnight incubation at 37 °C. The digested peptides were desalted on Sep-Pak C18 cartridge (Waters, Milford, MA, USA) and were completely dried under speed-vac.

### Mass spectrometry analysis

Peptides were resuspended in 50 *μ*L Solvent A (0.1% formic acid in water) and 3 *μ*L sample was loaded onto an analytic column (PepMap, 75 μm ID*50 cm 3 μm, ES803, Thermo Fisher Scientific) and separated with a linear gradient of 5–32% Solvent B (0.1% formic acid in ACN) for 70 min at a flow rate 300 nL/min. MS spectra were recorded on Q Exactive™ mass spectrometer (Thermo Fisher Scientific) interfaced with easy-nLC1000 (Thermo Fisher Scientific). The standard mass spectrometric condition of the spray voltage was set to 1.5 kV and the temperature of the heated capillary was set to 250 °C. The full scans were acquired in the mass analyzer at 400–1400 *m*/*z* with a resolution of 70,000 and the MS/MS scans were obtained with a resolution of 17,500 by normalized collision energy of 27 eV for high-energy collisional dissociation fragmentation. The advanced gain control target was 5 × 10^4^, maximum injection time was 120 ms, and the isolation window was set to 3 *m*/*z*. The Q-Exactive was operated in data-dependent mode with one survey MS scan followed by ten MS/MS scans, and the duration time of dynamic exclusion was 60 s. The mass spectrometry proteomics data have been deposited to the ProteomeXchange Consortium via the PRIDE^44^ partner repository with the dataset identifier PXD013966.

### Database searching and quantification

Collected MS/MS data were converted into mzXML files through the Trans Proteomic Pipeline (version 4.5) software and searched against the decoy UniProt human database (version 3.83, 186 578 entries) for the estimation of the FDR with the SEQUEST^®^ (version 27, Thermo Fisher Scientific) program in the SORCERER^TM^ (version 3.5, Sage-N Research, Milpitas CA, USA) search platform. Precursor and fragment ion tolerance were set to 10 ppm and 0.5 Da, respectively. Trypsin was chosen as an enzyme with a maximum allowance of up to two missed cleavages. Carbamidomethyl of cysteine (57.0215 Da) was considered as the fixed modification, while the variable modification was set for methionine oxidation (15.9949 Da). The Scaffold software package (version 3.4.9, Proteome Software Inc., Portland, OR, USA) was used to validate MS/MS-based peptide and protein identifications. Peptide and protein identifications were accepted if they could be established at greater than 95 and 99% probability, respectively, and if the protein identification contained at least two identified peptides with an FDR ≤0.1%. The MS1 intensity was measured using Scaffold Q+ (version 4.6.4, Proteome Software Inc., Portland, OR, USA). Normalized precursor ion intensities were acquired with 99% protein threshold, minimum of 2 peptides and 95% peptide threshold.

### Identification of DEPs and refinement of spectral count by MAI estimators

Relative protein quantitation was accomplished using spectral counting. Among identified 2,819 proteins, we excluded 40 keratins considering them as contamination, and 254 reverse phases then subsequent final 2,525 of identified proteins were identified. The normalized SC from triplicate datasets using scaffold was compared using PLGEM-STN to identify DEPs in MDA-MB468 grown under HG and GD conditions. The count values were fit to PLGEM, and DEPs were identified through a permuted STN test statistic^16^. The implementation was in R and used the PLGEM package in Bioconductor. We filtered statistically significant proteins using 0.01 as a p-value threshold. Then we refined SC of DEPs within the range of 0.01 ≤ p-value ≤ 0.05, those are excluded from first criteria p-value < 0.01. The refinement was made using MAI equation, $\widehat{{X}_{i}}=\,{W}_{i}\hat{a}+\bar{W}(1-\hat{a})$ ($\widehat{{X}_{i}}$ as the refined count, ${W}_{i}$ as the mis-measured observation, $i$ as repeated number of measures, and $\hat{a}$ as relation between potentially error-free covariates $\,{\sigma }_{x}$ and measured variable $\,{\sigma }_{w}$ in $\,{({\widehat{{\sigma }_{x}}}^{2}/{\widehat{{\sigma }_{w}}}^{2})}^{1/2}$ form). We computed error-free covariates $\,{\sigma }_{x}\,\,$as standard deviation of PLGEM calculated from the PLGEM linear regression model, $\mathrm{ln}(s)=k\,\mathrm{ln}(\bar{x})+c+\varepsilon$ (*s* and $\bar{x}$ as standard deviation and mean of repeated measures, *k* as the slope of regression line, *c* is intercept, and error term *ε*). The adjustment of SC was made when the plot of triplicated SC data exhibit skewness. The triplicated SC with skewness greater than 0 was regarded to be overestimated, skewness less than 0 to be underestimated and skewness equal to 0 to be truly estimated. We assume that ${W}_{1}\le {W}_{2}\le \ldots \le {W}_{n}$ and ${X}_{1}\le {X}_{2}\le \ldots \le {X}_{n}$. For two different conditions, the objective function is

$$\begin{array}{c}{\rm{s}}{\rm{k}}{\rm{e}}{\rm{w}}{\rm{n}}{\rm{e}}{\rm{s}}{\rm{s}}\,{\rm{o}}{\rm{f}}\,W > 0,\,\hat{{X}_{n}}={W}_{n}\hat{a}+\bar{W}(1-\hat{a})\,{\rm{a}}{\rm{n}}{\rm{d}}\,\,({X}_{1},\ldots \,,\,{X}_{n-1})=({W}_{1},\ldots \,,\,{W}_{n-1})\\ \,{\rm{s}}{\rm{k}}{\rm{e}}{\rm{w}}{\rm{n}}{\rm{e}}{\rm{s}}{\rm{s}}\,{\rm{o}}{\rm{f}}\,W < 0,\,\hat{{X}_{1}}={W}_{1}\hat{a}+\bar{W}(1-\hat{a})\,{\rm{a}}{\rm{n}}{\rm{d}}\,\,({X}_{2},\ldots \,,\,{X}_{n-1})=({W}_{2},\ldots \,,\,{W}_{n-1})\end{array}$$

The p-values and STN were re-computed using MAI estimator values by PLGEM-STN tool. Then we considered the recalculated p-value < 0.03 as statistically significant.

### Network analysis of 10 KEGG pathways and 5 metabolic processes

PPI network analysis was performed using Cytoscape program (version 2.8.2)^30^ and to assess the modeled PPI analysis, STRING (Search Tool for the Retrieval of Interacting Genes/Proteins) protein interaction database (version 10)^29^ was used. To display expression alternation of DEPs, Log2 fold change values were exhibited in two colors at the network plot: blue down-regulated DEPs, red for up-regulated DEPs. We categorized major PPI by 5 metabolic processes: nucleotide metabolic processes (nucleoside phosphate metabolic process, purine nucleotide metabolic process, pyridine-containing compound metabolic process, nucleotide biosynthetic process, ribonucleotide metabolic system), cellular respiration (aerobic respiration, respiratory electron transport chain, mitochondrial electron transport, NADH to ubiquinone), cellular amino acid metabolic process (alpha-amino acid metabolic process, cellular amino acid catabolic process, sulfur amino acid metabolic process), glucose metabolic process (glycolytic process, canonical glycolysis, gluconeogenesis), and fatty acid metabolic process (fatty acid β-oxidation, long chain fatty acid metabolic process, unsaturated fatty acid metabolic process, fatty acid biosynthetic process).

### Western blot validation

Fifty micrograms of proteins from each experimental group were applied to Bolt 4–12% Bis-Tris Plus gels (Invitrogen, Karlsruhe, Germany) and electrophoresed for 2 h 30 min at 80 V. Proteins were transferred onto a PVDF membrane in blotting buffer for 1 h at 100 V and blocked with 5% skim milk (Difco, Detroit, MI, USA) or 5% BSA (Gibco, Grand Island, NY, USA) in TBST for 1 h at room temperature. The blotted membrane was then incubated overnight at 4 °C with the different primary antibodies. Antibodies against GMPPA (1:4,000) and RRM2 (1:5,000) were purchased from Young in Frontier (Seoul, Korea), MAVS (1:5,000) was from Bethyl Lab (Montgomery, TX, USA), SOD1 (1:1000) and IPO4 (1:1000) were from Invitrogen (San Diego, CA, USA) and β-actin (1:10,000) was from Cell Signaling Technology (Beverley, MA, USA). Blots were then incubated with horseradish-peroxidase conjugated anti-rabbit IgG (GeneTex, Irvine, CA, USA, diluted 1:7,000 for GMPPA, 1:5,000 for MAVS and IPO4, Jackson ImmunoResearch, West Grove, PA, USA, diluted 1:10,000 for SOD1) and anti-mouse IgG (Jackson ImmunoResearch, West Grove, PA, USA, diluted 1:11,000 for RRM2) for 1 h at room temperature. Detection was performed using an ECL system (Amersham Pharmacia Biotech, Piscataway, NJ, USA).

### Correlation of EST and proteins

The expression levels of DEPs was assessed using EST database^37,38^. We used the Unigene EST profile (http://www.ncbi.nlm.nih.gov/UniGene), which is an approximate expression pattern inferred from EST counts and the cDNA library sources presented by health state, for the gene EST abundance in breast (mammary gland) tumor.

## Supplementary information

Supplementary Information

Supplementary Tables

## Data Availability

The MS data on MDA-MB468 is deposited in the ProteomeXchange under accession codes PXD013966. All reagents and relevant data are available from the authors upon request.
