# Supplementary material for: Refinements of LC-MS/MS Spectral Counting Statistics Improve Quantification of Low Abundance Proteins
Source: Sci Rep. 2019 Sep 20;9:13653. doi: 10.1038/s41598-019-49665-1 (PMC6754416; doi:10.1038/s41598-019-49665-1)
Supplement: Supplementary file 1 — Supplementary Information [file 41598_2019_49665_MOESM1_ESM.pdf]

Supplementary Information

# Refinements of LC-MS/MS Spectral Counting Statistics Improve Quantification of Low Abundance Proteins

Ha Yun Lee[1,+], Eunhee G. Kim [2,+], Hye Ryeon Jung[1], Jin Woo Jung [1],
Han Byeol Kim[1,3], Jin Won Cho[3], Kristine M. Kim[2,*], Eugene C. Yi [1,*]

[1]Department of Molecular Medicine and Biopharmaceutical Sciences, Graduate School of Convergence Science and Technology and College of Medicine or College of Pharmacy, Seoul National University, Seoul, 03080, South Korea

[2]Department of Systems Immunology, Division of Biomedical Convergence, College of Biomedical Science, Kangwon National University, Gangwon, 24341, South Korea

[3]Department of Integrated OMICS for Biomedical Science, Graduate School, Yonsei University, Seoul, 03722, South Korea

* Corresponding authors:

- Eugene C. Yi (E-mail: euyi@snu.ac.kr, Phone: +82-02-740-8986, Fax: +82-02-872-0630, Address: 103 Daehak-ro, Medical Science Building 305, Jongro-gu, Seoul 03080, Republic of Korea)
- Kristine M. Kim (E-mail: kmkim@kangwon.ac.kr, Phone: +82-033-250-8382, Fax: +82-033-250-8382, Address: 1 Kangwondaehak-gil, Biomedical Science Building B-202, Chuncheon 24341, Republic of Korea)

[+] These authors contributed equally to this work

This PDF file includes:

Supplementary Information S1.

Supplementary Information S2.

Supplementary Information S3.

Supplementary Information S4.

**Supplementary Information S1:** Script for MAI refinements after the PLGEM-STN analysis.

The text in bold can be replaced by user according to their sample information.

library(plgem)

library(Biobase)

# input directory to open file

dataFile <- **"/Users/junlee/Desktop/02. labelfree/test.txt"**

data <- read.table**("/Users/junlee/Desktop/02. labelfree/test.txt"**, sep='\t', header=TRUE, row.names = 1)

pData <- data.frame(CONDITION= c(**"CON","CON","CON","EXP","EXP","EXP"**))

# input replication time of analysis

replicate.number **<- 3**

# input total number of proteins

protein.number **<- 2525**

data.con <- data[1:protein.number,1:replicate.number]

data.exp <- data[1:protein.number,(replicate.number+1):(replicate.number*2)]

plgem.slope <- my.Eset.fit$SLOPE

plgem.intercept <- my.Eset.fit$INTERCEPT

```
# create a vector with random value
mean.con <-0
sd.con <-0
m3.con <-0
skew.con <-0
ln.mean.con <-0
esd.con <-0
mai.a.con <-0

mean.exp <-0
sd.exp <-0
m3.exp <-0
skew.exp <-0
ln.mean.exp <-0
esd.exp <-0
mai.a.exp <-0


# calculating MAI estimors
for (i in 1:protein.number)
{
  mean.con[i] <- rowMeans(data.con[i, ])
  mean.con[i] <- replace(mean.con[i], mean.con[i]<=0, 0.3333)
```

```
  sd.con[i] <- apply(data.con[i, ], 1, sd)
  sd.con[i] <- replace(sd.con[i], sd.con[i]<=0, 0.5774)
  m3.con[i] <-rowMeans((data.con[i, ]-mean.con[i])^3)
  skew.con[i] <- m3.con[i]/sd.con[i]^3
  ln.mean.con[i] <-log(mean.con[i])
  esd.con[i] <- exp(plgem.slope*ln.mean.con[i]+plgem.intercept)
  mai.a.con[i] <- sqrt(esd.con[i]^2/sd.con[i]^2)
  if (sd.con[i] > esd.con[i])
  {
    if(skew.con[i] >0)
    {
      data.con[i,data.con[i, ]>=max(data.con[i,])] <-
max(data.con[i,])*mai.a.con[i]+mean.con[i]*(1-mai.a.con[i])
    }
    else
    {
      data.con[i,data.con[i, ]<=min(data.con[i,])] <-
min(data.con[i,])*mai.a.con[i]+mean.con[i]*(1-mai.a.con[i])
    }
  }
}
for (i in 1:protein.number)
{
  mean.exp [i] <- rowMeans(data.exp[i, ])
  mean.exp[i] <- replace(mean.exp[i], mean.exp[i]<=0, 0.3333)
  sd.exp[i] <- apply(data.exp[i, ], 1, sd)
```

```
  sd.exp[i] <- replace(sd.exp[i], sd.exp[i]<=0, 0.5774)
  m3.exp[i] <-rowMeans((data.exp[i, ]-mean.exp[i])^3)
  skew.exp[i] <- m3.exp[i]/sd.exp[i]^3
  ln.mean.exp[i] <-log(mean.exp[i])
  esd.exp[i] <- exp(plgem.slope*ln.mean.exp[i]+plgem.intercept)
  mai.a.exp[i] <- sqrt(esd.exp[i]^2/sd.exp[i]^2)
  if (sd.exp[i] > esd.exp[i])
  {
    if(skew.exp[i] >0)
    {
      data.exp[i,data.exp[i, ]>=max(data.exp[i,])] <-
max(data.exp[i,])*mai.a.exp[i]+mean.exp[i]*(1-mai.a.exp[i])
    }
    else
    {
      data.exp[i,data.exp[i, ]<=min(data.exp[i,])] <-
min(data.exp[i,])*mai.a.exp[i]+mean.exp[i]*(1-mai.a.exp[i])
    }
  }
}

# merge by column
mai.esti.total <- cbind(data.con, data.exp)

# write cvs file
write.csv(mai.esti.total, "/Users/junlee/Desktop/02. labelfree/mai.csv")
```

**Supplementary Information S2:** Images of western blot analysis. Western blot analysis was performed on whole cell lysates from MDA-MB468 cells grown under the HG and GD conditions. GMPPA, RRM2, MAVS, IPO4 and SOD1 were detected and their relative expressions were correlated with the spectral count quantitative readouts. Western blot of a) GMPPA, b) beta-actin of GMPPA blot, c) RRM2, d) beta-actin of RRM2 blot, e) MAVS, f) beta-actin of MAVS blot, g) IPO4, h) beta-actin of IPO4 blot, i) SOD1 and j) beta-actin of SOD1 blot. Red arrow indicates the target protein and SOD1 samples were loaded in duplicate for each HG and GD conditions

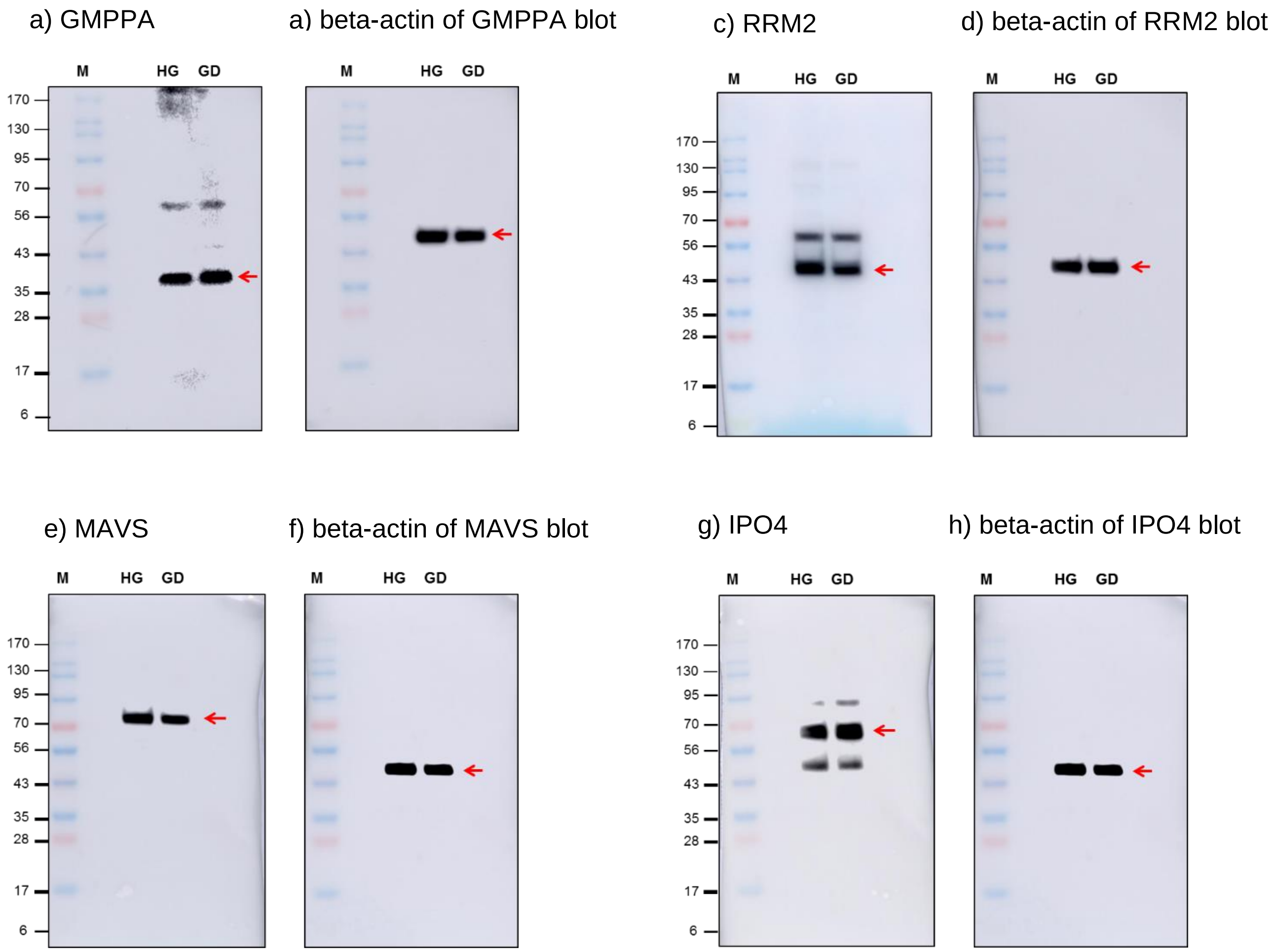

i) SOD1

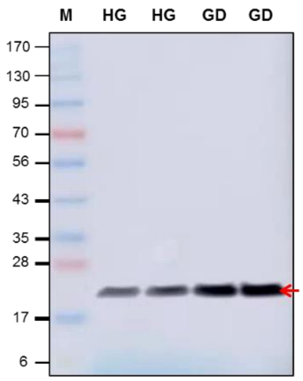


j) beta-actin of SOD1 blot

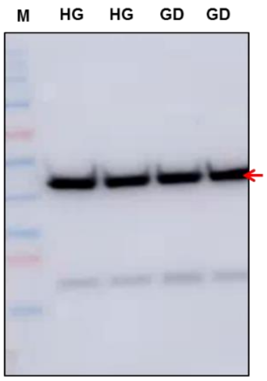

**Supplementary Information S3:** A plot of MS1-based intensity and spectral counts with R = 87.8%.

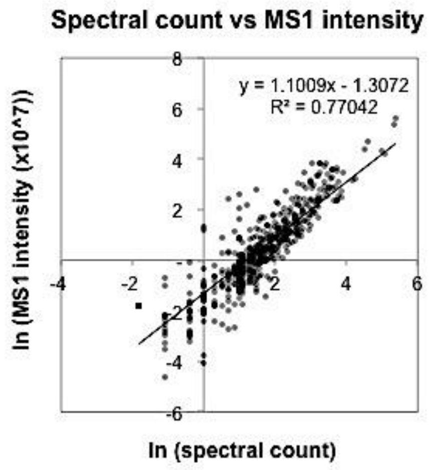

**Supplementary Information S4:** A plot of EST abundance of 269 MAI refined DEPs with 5 high-abundance DEPs (PKM, GAPDH, HSP90AB1, EEF2, HYOU1; SC >100 and PLGEM-STN p-value < 0.01) as reference group.

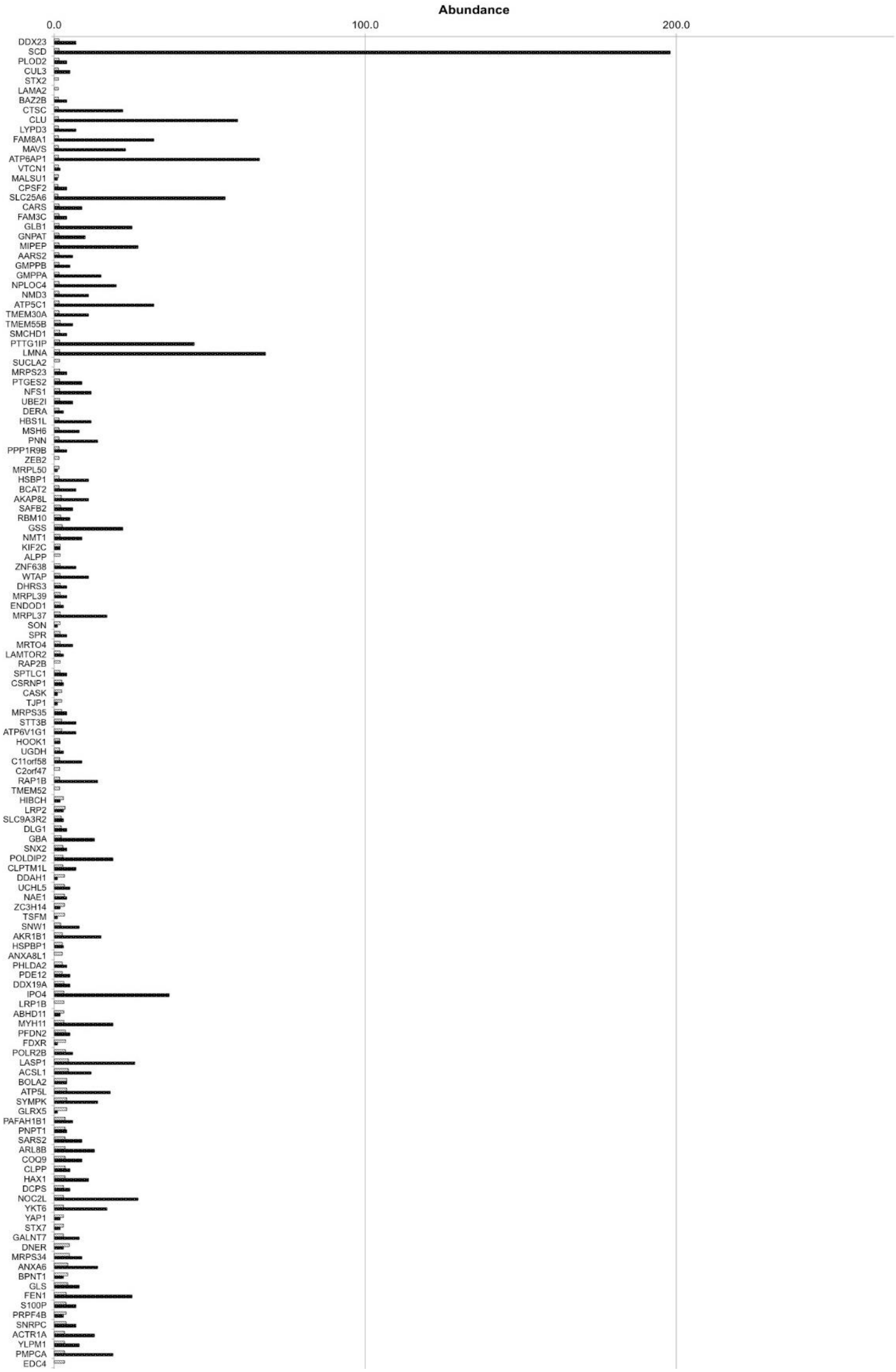

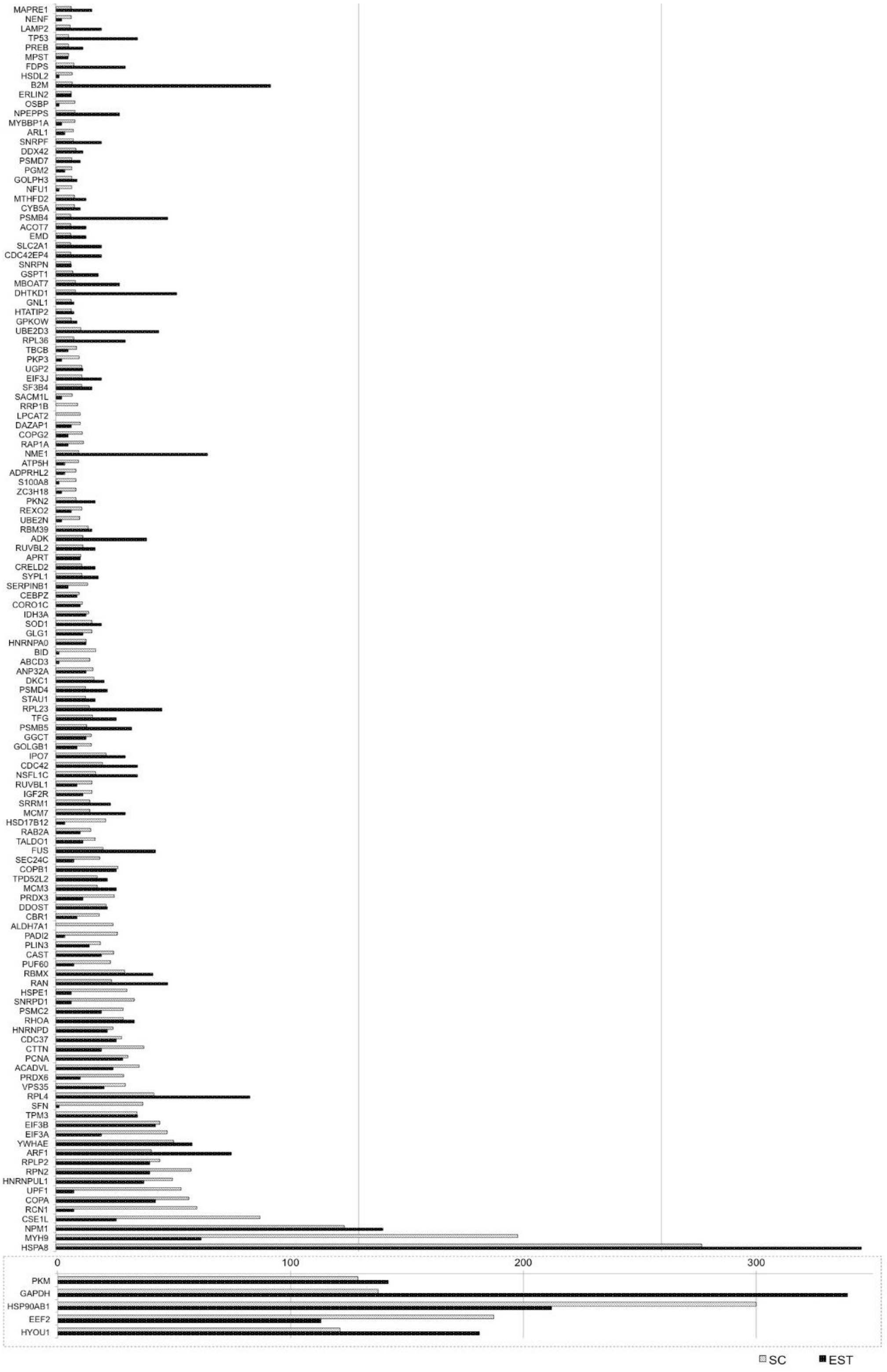
MAPRE1
NENF
LAMP2
TP53
PREB
MPST
FDPS
HSDL2
B2M
ERLIN2
OSBP
NPEPPS
MYBBP1A
ARL1
SNRPF
DDX42
PSMD7
PGM2
GOLPH3
NFU1
MTHFD2
CYB5A
PSMB4
ACOT7
EMD
SLC2A1
CDC42EP4
SNRPN
GSPT1
MBOAT7
DHTKD1
GNL1
HTATIP2
GPKOW
UBE2D3
RPL36
TBCB
PKP3
UGP2
EIF3J
SF3B4
SACM1L
RRP1B
LPCAT2
DAZAP1
COPG2
RAP1A
NME1
ATP5H
ADPRHL2
S100A8
ZC3H18
PKN2
REXO2
UBE2N
RBM39
ADK
RUVBL2
APRT
CRELD2
SYPL1
SERPINB1
CEBPZ
CORO1C
IDH3A
SOD1
GLG1
HNRNPA0
BID
ABCD3
ANP32A
DKC1
PSMD4
STAU1
RPL23
TFG
PSMB5
GGCT
GOLGB1
IPO7
CDC42
NSFL1C
RUVBL1
IGF2R
SRRM1
MCM7
HSD17B12
RAB2A
TALDO1
FUS
SEC24C
COPB1
TPD52L2
MCM3
PRDX3
DDOST
CBR1
ALDH7A1
PADI2
PLIN3
CAST
PUF60
RBMX
RAN
HSPE1
SNRPD1
PSMC2
RHOA
HNRNPD
CDC37
CTTN
PCNA
ACADVL
PRDX6
VPS35
RPL4
SFN
TPM3
EIF3B
EIF3A
YWHAE
ARF1
RPLP2
RPN2
HNRNPUL1
UPF1
COPA
RCN1
CSE1L
NPM1
MYH9
HSPA8
0
100
200
300
PKM
GAPDH
HSP90AB1
EEF2
HYOU1
SC
EST
